# diempy: fast and reference-free genome polarization and chromosome painting

**DOI:** 10.1093/g3journal/jkag140

**Published:** 2026-06-01

**Authors:** Derek Setter, Konrad Lohse, Stuart J E Baird

**Affiliations:** Institute of Ecology and Evolution, University of Edinburgh, Edinburgh EH9 3DW, United Kingdom; Institute of Ecology and Evolution, University of Edinburgh, Edinburgh EH9 3DW, United Kingdom; Institute of Vertebrate Biology, Czech Academy of Sciences, Brno 603 65, Czechia

**Keywords:** genome polarization, reference-free, admixture, genetic barriers, chromosome painting, population genomics

## Abstract

Most ancestry inference methods rely on putatively pure reference panels to define ancestry informative variants. This approach is often unrealistic and can bias inference. The genome polarization algorithm *diem*, introduced previously by Baird et al., avoids reference panels by jointly inferring the polarity of common allelic states and quantifying variant diagnosticity via an expectation–maximization procedure. Importantly, we use “polarization” strictly to mean the assignment of alleles to opposing sides of a barrier to gene flow, rather than the assignment of ancestral versus derived states. Here, we present diempy, an efficient python implementation of *diem* coupled with tools that turn polarized calls into analysis-ready outputs. diempy offers lossless VCF-to-*diem* BED conversion; ploidy-aware handling of individuals and chromosomes; flexible masking of sites, regions, and individuals; and interactive visualization of polarized genomes, hybrid indices, clines, and ternary plots. Postprocessing functions include thresholding via the diagnostic index, kernel smoothing, and automatic detection and run-length encoding of contiguous ancestry tracts. BED-based I/O facilitates integration with population-genomic workflows (e.g. filtering by annotation or ploidy). These features make reference-panel-free genome polarization with diempy practical and reproducible for studies of population structure, admixture and species barriers.

## Introduction

Analyses aimed at understanding population structure, hybridization and admixture often start by estimating and visualizing the ancestry contributions in a sample of genomes. In the simplest case of two ancestry sources, individual diploid genomes are mosaics of tracts that are homozygous for either source or heterozygous by source.

While numerous tools exist to analyze these mosaics, they generally fall into categories that require substantial *a priori* assumptions. For example, traditional clustering algorithms such as ADMIXTURE (Alexander et al. 2009) and STRUCTURE (Falush et al. 2003) efficiently estimate overall ancestry proportions, but providing high-resolution estimates regarding exactly *where* that ancestry lies along the genome requires computationally intensive linkage models. Conversely, distance-based analyses (e.g. local PCA) can generate local ancestry estimates, but require the user to arbitrarily define window sizes and clustering thresholds *before* analysis—a counterproductive requirement when exploring a highly variable genomic landscape where the scales of selection and admixture are unknown.

Furthermore, methods that utilize sequence pattern-matching or local ancestry inference fundamentally rely on predefined reference panels to define “ancestry informative markers” (Schumer et al. 2020). Although for some taxa and sampling schemes a clear *a priori* distinction between putative source and admixted populations is possible (Li et al. 2022; Heidbreder et al. 2026), reference panels/populations are unlikely to exist for most taxa that experience admixture. Moreover, declaring a reference panel as “pure” introduces a well-documented bias against the detection of introgression (Gompert et al. 2017). For example, in taxa that meet at a narrow hybrid zone, ancestry inference based on reference individuals sampled outside of the hybrid zone is by definition blind to introgression that has escaped the hybrid zone (Ebdon et al. 2025).

The *diem* genome polarization algorithm (Baird et al. 2023) avoids these limitations by replacing parameter-rich equilibrium models and reference panels with a core population genomic concept: a barrier (*sensu* Barton and Bengtsson 1986) to gene flow. We stress that throughout this article, we use the term “polarization” strictly to describe the assignment of alleles to either side of this barrier. It should not be confused with the traditional population genetic usage for estimating the ancestral state of a polymorphic site. For similar reasons, when we use the term “reference” we refer to reference panels rather than the reference genome assembly used for read mapping, the genotypic state of which forms no part of *diem* inference. By jointly inferring the barrier-based polarity of common allelic states and quantifying variant diagnosticity via an expectation–maximization (EM) procedure, *diem* allows the scale of ancestry tracts to emerge *post hoc* from the data itself. *diem* is not the only algorithm that can do this: the SiteBySite extension of the STRUCTURE framework does so without reference panels (Falush et al. 2003). However, STRUCTURE’s explicit model of population genetic equilibrium clusters means this linkage algorithm does not scale to genomic applications. Furthermore, while comparison over a small genome interval showed strong consistency between SiteBySite and *diem* estimates (Baird et al. 2023), SiteBySite output interpolates state over missing data regions, leading to local sharp contrasts with evidence-based *diem* inference.

The core EM algorithm for genome polarization is efficient, deterministic and has been implemented in Mathematica, python, and R, Baird et al. (2023), with a current R CRAN package (Baird et al. 2026). Genome polarization has been used in analyses of whole genome sequence and reduced representation data to investigate species barriers and adaptive variation that penetrates them across a wide range of systems (Baird et al. 2023; Giakoumis et al. 2023; Ebdon et al. 2025; Petružela et al. 2025). The flexibility and generality of reference-panel-free genome polarization avoids the need for any arbitrary prefiltering of data and instead gives users the ability to explore and visualize polarized data for a range of diagnosticity thresholds and smoothing decisions.

Here, we present diempy, an efficient Python implementation of *diem* coupled with a suite of tools designed to turn polarized calls into analysis-ready outputs. Built on a robust, object-oriented architecture, diempy integrates seamlessly into standard population genomic pipelines, allowing researchers to investigate genome-wide barriers to gene flow quickly, transparently, and entirely free from the biases of reference panels.

## Methods

diempy implements the EM algorithm for genome polarization of Baird et al. (2023) as an open source software package in Python 3 (Van Rossum and Drake 2009) (version ≥3.9). The source code is available on GitHub (https://github.com/Studenecivb/diemPy), and the package is available on PyPi for direct installation using pip (https://pypi.org/project/diempy/). Below, we describe the input data, provide a brief review of the EM algorithm and its outputs, and give an overview of the software implementation.

### Data input and limitations

To perform genome polarization using diempy, the user provides a VCF file containing genotype data for all individuals, alongside the physical length of each chromosome/contig in base pairs within the header. Since the *diem* algorithm is agnostic to the physical position of variants, the VCF input need not be aligned to a chromosome-level assembly. The term “chromosome” is used here to broadly denote a known linkage group, which could be a “scaffold” or “ddRAD interval.” The raw data are then preprocessed using the vcf2diem command-line function, which losslessly compresses and formats the data for analysis.

The fundamental unit of analysis in diempy is the genotypic state of a variant. Because the algorithm is strictly reference-panel-free, it cannot rely on imputation or linkage disequilibrium models to infer missing data. Instead, the EM algorithm organically copolarizes variants by identifying correlations across the provided genomes. Consequently, accurate genome polarization relies on the confident identification of homozygous states across individuals. Ideal input data consist of high-confidence genotype calls, typically requiring moderate to high sequencing coverage (e.g. >10x for whole-genome sequencing) or robust reduced-representation data (e.g. RADseq or targeted capture).

However, lower coverage data simply adds unbiased (assuming no statewise coverage bias with respect to the reference genome) input noise to *diem*: true HET states are called as HOM state. Sites where HET states are stochastically miscalled as HOMs reduce the power of *diem* without changing its convergence expectations and will simply have low diagnostic value. Where a genome wide bias exists, e.g. reads from one side of a barrier tend to map better to the reference genome, HET introgression to that side will be underestimated. This bias can be minimized by mapping to an outgroup to the analyzed genomes with equal expected distance to all samples. As that distance increases, the frequency of miscalled HOMs will increase due to divergence-loss in coverage, but once more in an unbiased way.

### Algorithm overview and genome polarization

The core function of diempy is to polarize genomic data with respect to a barrier to gene flow without relying on predefined, reference panels. We define “polarization” strictly as the assignment of alleles to either side of a barrier, which should not be confused with determining the ancestral state of an allele. To achieve this, diempy implements the EM algorithm *diem* (Baird et al. 2023). The algorithm seeks to label the two most common alleles of each variant such that the genome-wide correlation of allelic states across individuals is maximized. For a dataset of *n* individuals and *m* variants, each variant $M_{i}$ is encoded into four possible genotypic states: homozygous for the first common allele, heterozygous, homozygous for the second common allele, or unencodable/missing data (0,1,2,U, respectively). The EM procedure begins with a state of ignorance, assigning a random “null” polarity $\phi_{i}\in \{0,1\}$ to each variant. The algorithm then proceeds iteratively through two main steps:

1. *Expectation step*: given the current polarities, the algorithm calculates a hybrid index (HI) for each individual. The HI represents the proportion of an individual genome’s alleles that are associated with one side of the barrier.
2. *Maximization step*: given the current hybrid indices across all individuals, the algorithm evaluates every variant independently. It calculates the likelihood of the variant’s encoded state under both possible polarities (ϕ=0 or ϕ=1). The polarity that yields the higher likelihood is selected for the next iteration.

These steps are repeated until the polarities converge on a stable labeling, effectively polarizing the genomes and identifying the strongest barrier to gene flow within the sampled individuals.

### The diagnostic index

A key output of the *diem* EM algorithm is the diagnostic index (DI). The DI of a variant quantifies how well its configuration aligns with the consensus genomic barrier identified by the algorithm. Mathematically, the likelihood $L(M_{i}|\phi )$ of observing a variant $M_{i}$ under a specific polarity *ϕ* is calculated conditional on the expected frequencies of the genotypic states across all other variants in the dataset. The DI (${\text{DI}}_{i}$) for the *i*th variant is defined as the maximum log-likelihood of the variant’s state across its two possible polarities:


$${\mathrm{DI}}_{i}=\max_{\phi \in \{0,1\}}\ln\;L(M_{i}|\phi ,I_{-i},\epsilon )$$

where $I_{-i}$ represents the polarized state counts of all other variants (excluding *i*) and ϵ determines the degree to which the model tends to the diagnostic ideal. Variants with high DI values carry strong information regarding the barrier separating the genomes, indicating that individuals on one side are predominantly homozygous for one allele, while individuals on the other side are homozygous for the alternative allele. Conversely, variants with low DI values are uninformative with respect to the barrier and may represent shared ancestral variation, incomplete lineage sorting, or mapping/genotyping errors.

### Software implementation and workflow

The diempy package implements the *diem* EM algorithm in Python 3, designed for seamless integration into broader population genomic pipelines. During the core EM polarization, thresholding, and smoothing steps, the software efficiently handles data as a matrix of states, where rows represent individuals and columns represent their corresponding genotypic state at each variant. A high-level overview of the diempy analysis workflow encompasses four primary stages:

1. *Data import and preprocessing*:
  The package offers lossless conversion of VCF files to *diem*-formatted BED files using the vcf2diem tool. This stage handles chromosome definitions and ploidy-awareness. Following this initial conversion, users can flexibly mask specific sites, regions, or individuals to refine the dataset prior to analysis.
2. *Polarization*: The complex steps of initialization and optimization are handled automatically. By simply calling the polarize() function on the data object, diempy automatically initializes a random null polarity, runs the core EM algorithm to convergence, and calculates the DI and support values for all variants.
3. *Post-polarization processing*: Users can filter less informative variants via DI thresholding and apply Laplace kernel smoothing to minimize localized noise. In downstream analyses, the state matrix is compressed into a lossless reduced representation, recasting the data for each sample as contiguous tracts of shared state (U,0,1,2). A major strength of the *diem* approach is that these tracts naturally emerge from the data rather than being directly inferred. These tracts are stored sequentially for each individual along each chromosome. Note that we use the general term “contig” for the collection of ancestry tracts for a single individual and contig/chromosome in the VCF.
4. *Exploration and export*: The package includes tools for the interactive visualization of polarized genomes, hybrid indices, and genomic clines. Furthermore, BED-based I/O facilitates the straightforward export of the emergent tracts for subsequent downstream applications.

Comprehensive API documentation detailing the underlying data structures and specific method calls is maintained on ReadTheDocs (https://diempy.readthedocs.io) alongside an interactive tutorial for standard analysis workflows.

### Benchmarking

We use simulation in msprime (Kelleher et al. 2016) to benchmark the performance of diempy with respect to speed, memory use, and accuracy. In all cases, we use a secondary contact model in which two populations split from a common ancestor $T_{\mathrm{split}}$ generations ago, then exchange migrants at some time in the more recent past. For testing speed and memory use, the performance is invariant to the underlying model; only the number of markers and sample size matters. We generated a dataset with m=1,000,000 variants an n=1,000 individuals for a total of $10^{9}$ data points, subsampling this to benchmark run time as a function of the number of markers and number of individuals, and we compare the performance for serial processing on k=1 core to that with parallel processing on k=5 and k=20 cores. We also compare the performance of this implementation of *diem* to a previous implementation in Mathematica (Wolfram 2025) using two real-world datasets: one with m×n=2.3 million variants by 250 genomes and the other m×n=15.8 million variants by 128 genomes. Again, the performance depends only on the data size and not the underlying biology.

In addition to benchmarking, we analyzed the power of the *diem* algorithm to recover signatures of introgression. Here, the underlying model *does* matter. We consider two diploid populations, the *donor* and *recipient*, that split without migration from a common ancestor population $T_{\mathrm{split}}$ generations in the past, with uni-directional migration starting 100 generations ago at a rate of $2N_{e}m=0.5$ from the *donor* to the *recipient* (forward in time). All populations have size $N_{e}=1,000$, and we vary the split time $T_{\mathrm{split}}\in \{2N_{e},4N_{e},8N_{e}\}$. We consider a single 200-Mb chromosome with recombination rate $\rho =10^{-9}$ and mutation rate $\mu =10^{-7}$ to generate a dense set of variants. We use an infinite sites mutation model and record migration events to clearly identify introgressed tracts. We measure the *recall* and *precision* of identifying introgressed variation within the recipient population as a function of the DI. Here, a true positive is a variant in any individual that is held within an introgressed tract (regardless the state) and we aggregate these calls across all individuals. *Recall* is defined by the the number of true positive calls in the subset of markers retained after thresholding relative to the total number true positives in the full dataset. *Precision* is defined as the true positive calls in the subset relative to the total number of variants identified as introgression, again aggregated across individuals.

### Dependencies

diempy has the following dependencies: numpy ≥1.20.0 (Harris et al. 2020), pandas ≥1.3.0 (McKinney et al. 2010; The Pandas Development Team 2022), numba ≥0.56.0 and ≤2.3 (Lam et al. 2015), matplotlib ≥3.4.0 (Hunter 2007), docopt ≥0.6.2 (Keleshev 2012), scikit-allel ≥1.3.0 (Miles et al. 2024), joblib ≥1.5.3, ipympl ≥0.10.0 (Hunter 2007), and pysam ≥0.24.0.

## Results

### Example analysis

We provide an overview and extensive API documentation of the software (https://diempy.readthedocs.io), as well as an illustrative jupyter lab tutorial and workflow (https://github.com/DerekSetter/tutorial-DiemPy/). We strongly recommend using this tutorial as a starting point for diempy analysis.

Here, we illustrate the step-by-step use of diempy using the example dataset provided with the tutorial. The example dataset comprises whole genome sequence data for 20 individuals from a sister species pair of Scarce Swallowtail butterflies: *Iphiclides podalirius* and *Iphiclides feisthamelii*. The data include individuals sampled throughout the range of each species as well as six individuals from the hybrid zone of the two species. Analyses of the full dataset (including *diem* polarization) are described in detail in Ebdon et al. (2025). The example data comprise a subset of 2,000 consecutive variants from each of the 30 chromosomes (about 0.13% of the total data).

#### Data and preprocessing

As described above, the example data are preprocessed using the vcf2diem function. This function performs a lossless compression, and simultaneously formats and filters the data for use in diempy. Not all variants included in the input VCF file are passed to *diem*: to be accepted, the two most common alleles (A,B) of a variant must both appear in the homozygous state in at least two individuals in the dataset. Within accepted variants, missing genotype calls and genotypes involving rarer alleles give rise to “U” genotypes within diempy, that is: not encodable with respect to (A,B) but still permissible for *diem*. For each accepted variant, the number of alleles, and whether those alleles occupy one base or a variable number of bases (indels) is recorded in the *diem_input.bed* file. Rejected variants are recorded in the *diem_excluded.bed* file with the reason for their exclusion, allowing users to explore relaxed filtering by moving variants from excluded to input using bedtools (Quinlan and Hall 2010). The **vcf2diem** data compression is lossless up to the ten most common alleles of a variant.

The third output of **vcf2diem** is a *diem_meta* file. Each row corresponds to a chromosome/contig in the input VCF. Columns record the start and end positions of chromosomes/contigs, the location of the first and last variants used for polarization, the total number of variants retained per chromosome/contig, and their relative recombination rates (defaulting to 1.0). Each remaining column specifies an individual and its *ploidy* with respect to each chromosome. In combination with the generalized homogeneous/heterogeneous *diem* state definitions (Baird et al. 2023) this allows arbitrary ploidies to be encoded (e.g. multiple hemizygous sex chromosomes or a mix of sexes sampled in a haplodiploid system). Because VCF files do not contain such meta information, by default, **vcf2diem** sets all ploidies to 2. While the *diem* algorithm is largely robust to errors in ploidy, corrected ploidies should be entered by the user. For example, in an X-Y system, males are haploid for the X chromosome, so their ploidy for the X should be set to 1. To correct ploidy information, the user must supply a tsv file in which the first column lists individuals in the dataset (header *#Inds*) and each remaining column corresponds to a chromosome/contig which has nondefault ploidy (headers must match the chromosome/contig names in the input VCF file). From within diempy, the meta data file can be updated using the diem.update_meta() function (the online tutorial provides an example).

#### Polarization

**Fig. 1.**
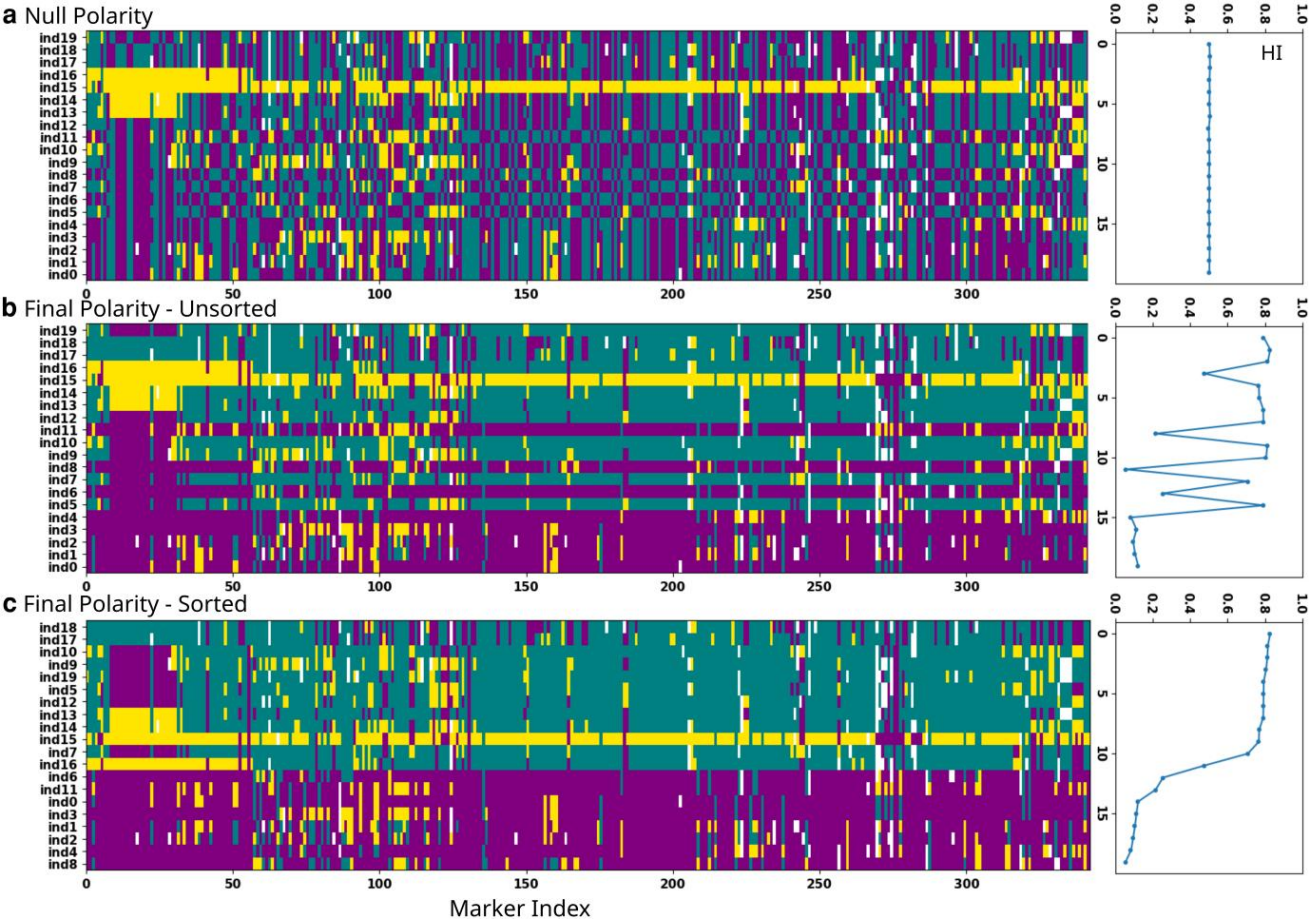
Polarizing variant data. The state of each individual is plotted for each variant indexed along the genome (i.e. the physical distance between variants is ignored). Purple and cyan correspond to the homozygous allele assignment that distinguishes the two sides of the barrier. Yellow corresponds to heterozygous states, and white corresponds to the U state (missing, or involving rare alleles). The HI of each individual is plotted to the right of the painting. (a) The null polarity with alleles randomly assigned. (b) The genotype states after polarizing. (c) The polarized data with individuals sorted by *diem*-estimated HI. Note that individual 15 is largely heterozygous in this subset of the data but not positioned at the center of the barrier. This is because HI values are calculated across the entire genome and this individual is generally less admixed in other chromosomes.

To perform polarization, the data are loaded into diempy as an instance of the DiemType class. Internally, polarization occurs in two steps. First, the HOM data labels are flipped to a random null polarity. This removes any biases about the assignment of alleles to a parent population or species. For example, genotype calls in a VCF file are generally polarized with respect to the allelic state of the reference assembly which may be estimated from an arbitrary individual/haplotype (which may be included in the set of genomes being analyzed), a consensus sequence across many individuals or a sample from an outgroup. An example of a null polarity can be seen in Fig. 1a. Under null polarization, the estimated HI of each individual will be approximately 0.5. Second, the *diem* algorithm seeks to maximize the difference between individuals by placing them on either side of a *barrier* to gene flow identified as the biggest step in estimated HI of the (sorted) individuals. It does so by iteratively checking the labeling of each variant, flipping the label when it increases the likelihood of the data given the current barrier conformation. The algorithm is deterministic in that a given null polarity (which can be input) results in one and only one polarization.

The output is a maximum likelihood labeling of the data that we visualize in Fig. 1b. We can distinguish the barrier more clearly by resorting individuals by HI (Fig. 1c). The polarized data can be stored directly as a DiemType object, which is efficient and useful for further analyses within diempy, or as a BED file which has less efficient storage but allows access for downstream analysis outside of diempy.

#### Thresholding

**Fig. 2.**
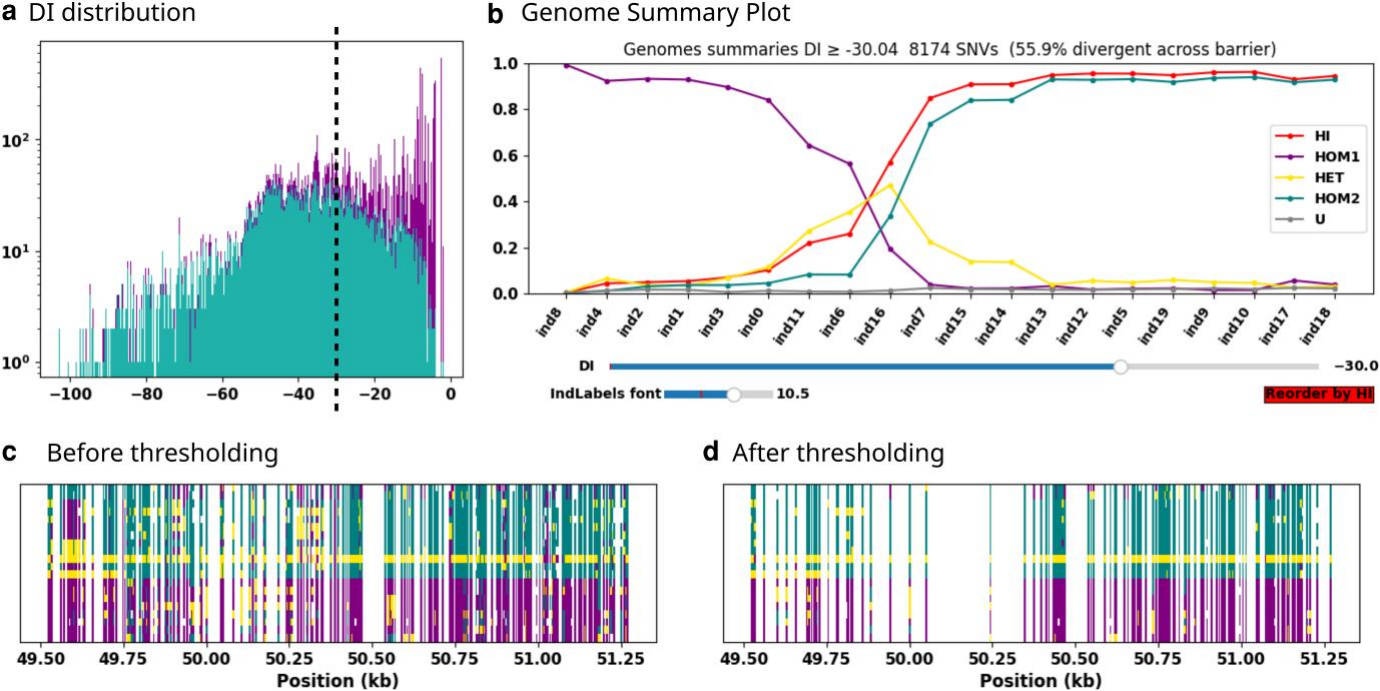
Applying a DI threshold. (a) The distribution of DI values. Cyan shows the counts of the unique variant configurations in 500 bins of DI values, with high values (closer to 0) corresponding to high diagnosticity. For the set of variant configurations in a given bin, the magenta bar indicates how many times those configurations appear in our dataset. (b) Four summaries of the data for each individual: the HI value, the proportion of “U” (Unencodable) genotypes, and the proportion of genotypes homozygous for the two alternative alleles. (c) The same data from Fig. 1 with variants in their physical positions and separated by whitespace. (d) The variants remaining after removing sites with DI value ≤−30.

For polarized data, diempy returns the DI for each variant, a measure of how well its configuration matches the identified genetic barrier. A variant configuration is simply a vector of genotypic states across individuals, corresponding to a given column of the painting in Fig. 1c. Note that many variants in the dataset will have the same configuration. We first consider the DI distribution, placing the set of unique configurations in the example dataset into 500 bins (Fig. 2a, cyan). The right-most bin contains the unique variant configuration that best fits the barrier and has highest DI, i.e. all individuals to the left of the barrier are homozygous for one allele while all individuals to the right of the barrier are homozygous for the alternative allele. In magenta, we show the counts of the variants in each of the cyan bins. For example, the most diagnostic variant configuration appears 10 times in the example dataset.

Variant configurations with low DI values carry little information about the barrier that separates samples and may correspond to ancestral shared variation, genotyping errors or introgressed variation that has risen to very high frequency. Thus, it may be useful to remove those variants from downstream analysis. This can be achieved using the *threshold* function in diempy. The choice of DI threshold will be unique to each dataset and should be informed by the DI distribution and the research question. We provide the interactive GenomeSummaryPlot() (Fig. 2b) to explore how thresholding affects the data. For example, here we see that retaining variants with DI≥−30 yields a strong separation, with the peripheral individuals achieving HI values close to 0 and 1. Of course, this threshold makes the retained data more sparse, as is evident in Fig. 2c and d, where variants are plotted at their relative position in the chromosome. It is worth remembering that even without DI thresholding, variant densities in population genomic data are likely to be sparse and uneven.

#### Smoothing

After thresholding, the polarized genotypes of an individual may consist of long tracts with (mostly) identical genotype state. In particular, we may be interested in tracts of admixture which, when rare, appear as consecutive runs of variants that are heterozygous by source (e.g. the central individual in Fig. 2c). The distribution of the lengths of these tracts is open to inference (Ebdon et al. 2025) and leads to very compact genome representations. However, on closer inspection, obvious blocks often contain tiny imperfections that would break up automated tract length measurement. One solution is a low-frequency-pass filter that removes any high-frequency changes in state along the chromosome. We implement this using kernel smoothing.

**Fig. 3.**
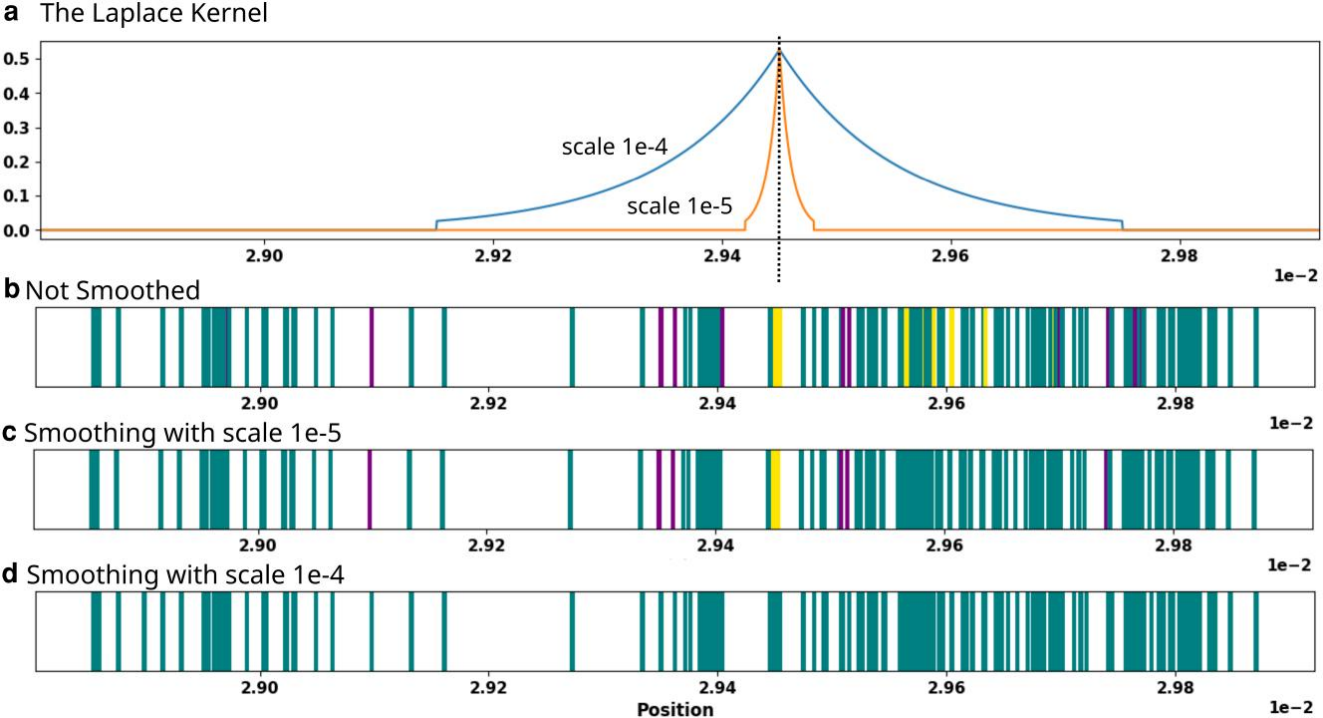
Smoothing polarized data with a Laplace kernel. The variants are plotted on a unit scale obtained by normalizing the base pair positions by the chromosome length. (a) The (truncated) Laplace kernel centered on the indicated variant for two different scale values. (b) The genotype of the second individual from the top in Fig. 2d. Panels (c) and (d) show the effect of smoothing this genotype using the two different scales.

We smooth the genotypes of each individual separately. For a given individual, we trace along the chromosome and smooth each site independently. To smooth a site, we assign a weight to it, as well as to the sites in its vicinity. We calculate the total weight of each possible state in this region and assign the focal site the state with the highest weight. The state may change, or it may remain the same. Here, we use the Laplace Kernel to assign weights (Fig. 3a).

The Laplace Kernel is a reflected exponential distribution and a natural biological choice: recombination diminishes the genealogical association between variant sites in the genome: given a random point in the genome, the distance to the next recombination breakpoint in the history of a sample is exponentially distributed. Thus, a Laplace kernel more accurately models the expected decay of LD with physical distance than, for example, a Gaussian kernel, which assumes a squared-distance decay. Furthermore, because the appropriate smoothing scale depends on the specific biological history of the dataset (such as the time since admixture and species-specific recombination rates), it must be parameterized by the user. We note that by default, diempy assumes that the total recombination rate of each chromosome is identical. While this is true in some organisms, users must adjust this default to reflect the recombination dynamics (e.g. differences in recombination rate among autosomes or a difference in the scaled recombination rates between autosomes and sex chromosomes) of their study system (see tutorial).

What remains is to choose the appropriate smoothing scale that determines the total breadth of the Laplace distribution. In Fig. 3a, we show the Laplace kernel with two different scales used to smooth the haplotype in Fig. 3b. For the smaller scale (1e−5), many of the small tracts have been smoothed over, but larger tracts, such as the one surrounding our focal heterozygous genotype, remain (Fig. 3c). By increasing the scale of the Laplace distribution, we start to smooth over larger tracts as well (Fig. 3d). Note that for computational efficiency, we truncate the Laplace distribution at a distance where the contribution of a given site becomes negligible.

In summary, the appropriate choice of the Laplace scale will be unique to each dataset and research question. In the online tutorial, we provide guidance for testing and choosing among different scale values, e.g. by measuring the number of sites that change for different values.

#### Analyzing tract lengths

Once we have applied DI thresholding and/or smoothing to the polarized data, individual genomes can be represented as a set of tracts of contiguous ancestry. The length distribution of these tracts across a sample is highly informative, for example, about the rate and timing of admixture (Baird 1995; Ebdon et al. 2025). To access this information directly, we apply the create_contig_matrix() function to our DiemType instance. This returns a run-length encoding of genotypes as a contig—an ordered list of contiguous intervals of identical state with end points defined by the position of the first and last variant that define the tract. In the tutorial, we demonstrate how to construct contigs, plot the length distribution of ancestry tracts, and save this information in BED format.

#### Browsing the data

Genome polarization data are typically very rich. We provide interactive visualizations that summarize data along multiple axes, making previously published styles of plot available publication-ready. Interactive “DI sliders” allow the user to examine the consequences of different thresholding levels. A global view of all sites in the data forms a circular “iris plot” which may show that barrier sites (maximum DI) are over-represented on certain chromosomes (e.g. sex chromosomes, Ebdon et al. 2025) or restricted to particular genomic regions (e.g. a polymorphic inversion in an otherwise unstructured population). Plots of polarized chromosomes may also reveal segregating introgression tracts and their shared boundaries, Fisher junctions (Fisher 1949, 1954) across individuals. All feature plots are browsable: placing the cursor over a feature reports its genome location in the coordinates specified in the VCF (and the individual(s) in which it occurs).

#### Masking

It is rare that a first analysis is final. An initial genome polarization may reveal outlier genomes or genomic regions that are not relevant to the barrier of interest. Rather than restarting the *diem* workflow from scratch, masking allows the user to refine their analyses mid-flow. Crucially: masked data are still polarized by *diem*, but have no influence over that polarization. For example, an outlier individual incorrectly included from a sister taxon can be masked to remove its influence (without constructing a new VCF file), while being polarized to show potential introgression relevant to the barrier of interest.

On the along-genome axis, a region of low effective recombination (little change in polarized state across many sites) may correspond to a trans-species polymorphic inversion. While the inversion is its own (old) local barrier, the user may not wish to confound its effects with recent (speciation) barrier signal. Once the inversion is identified by an initial polarization, it can be masked for an iterative *diem* analyses, which may reveal barriers associated with the more recent species divergence. Similarly, mitochondria and nonrecombining Y,W chromosomes should be masked: polarization will then highlight sites which differentiate variation in these nonrecombining compartments across the focal barrier. While each system and question is likely to have its own optimal masking, finding it is computationally straightforward given the BED file workflow and efficient (re)polarization step.

### Benchmarking

#### Speed

To benchmark the performance of diempy, we simulated a recent pulse of admixture between two divergent populations using msprime (Kelleher et al. 2016; Baumdicker et al. 2022) (see Methods). Note that while this demography is apropos of barrier analysis, the benchmarking results are model invariant, i.e. only the size of the dataset matters. We record the run time for a single expectation maximization step of the algorithm with varying number of samples and markers in Supplementary Fig. 1.

Run time increases linearly with the number of variants regardless of the number of samples or cores used (Supplementary Fig. 1). However, we observe some overhead cost to parallel processing when the number of variants is small. In contrast, there is a sublinear cost to increasing the number of samples: a 10-fold increase in the number of samples from n=10 to n=100 increases run time by approximately 1.2-fold, while a 100-fold increase in sample size (to n=1,000) induces only a 2.8-fold increase in run time. Parallelization reduces the run time but with diminishing returns: k=5 cores yield an approximately 5-fold reduction in run time while k=20 cores only provides an approximately 10-fold reduction. The memory requirements for both the sequential and parallel versions scale with the size of the state matrix: diempy requires approximately 12 GB of memory for m=1,000,000 variants and n=1,000 individuals, i.e. $10^{9}$ data points.

Previous implementations of the *diem* algorithm in three different programming languages shared very similar performance profiles to each other (Baird et al. 2023). This is unsurprising, given the computational simplicity of the algorithmic core, and the fact that all three implementations used the same grain of parallelization (Kruatrachue and Lewis 2002). Prompted by the design criterion of fitting seamlessly into bedtools workflows, diempy uses a different (finer) grain of parallelization, and is the first implementation to differ significantly in performance. We tested diempy against one of the coarse grained implementations: diemMca, implemented in Mathematica (Wolfram 2025). For a realworld dataset of m×n=2.3 million variants by 250 genomes diempy converged to the same labeling >12 times faster than diemMca (2 vs 25 min), this using 8 cores of the same 2021 laptop, with the diemMca input divided into 8 parallelization chunks. For a second realworld dataset of m×n=15.8 million variants by 128 genomes diempy converged >17 times faster than diemMca (10 vs 171 min), with the diemMca data divided into 32 chunks. We cannot claim this speed up was entirely by design—we did not analyze the parallelization grain size trade-off (Kruatrachue and Lewis 2002) *a priori*, and then optimize. Rather, it appears that the smoothed workflow and “Pythonic” design criteria lead naturally to a granularity better suited to the task, with increasing diempy efficiency benefits as more variants are considered. The efficiency gain is not a free lunch. By increasing the number of parallelization chunks in coarse-grained *diem* (e.g. 32 vs 8), its memory footprint can be controlled, leaving the task size open ended (given time is not an issue). This flexibility is sacrificed in diempy: maximum task size is now bounded by available RAM, but this is a concern that has rapidly diminished even since course-grained *diem* was made available in 2023. Data exceeding laptop constraints can either be processed with coarse grain or outsourced to a high-RAM cluster.

#### Power

The *diem* algorithm seeks to label variants by origin with respect to a gene flow barrier. In so doing, the variants with the highest DI value are those which best separate individuals into two groups by HI and serve as the “background” in which we can observe introgression. Generally, these *ideal* variants are those for which all individuals are homozygous for the allele assigned to their respective side of the barrier. The *nonideal* variants postpolarization correspond to migration, incomplete lineage sorting (ILS), or high-frequency local (i.e. population-specific) polymorphism. In the *diem* model, cross-barrier polymorphism due to ILS or local variation tend to have lower DI values than those due to migration. By setting a threshold on the DI for downstream analyses, we hope to retain only those variants which truly represent migration events such that the emergent ancestry tracts truly represent introgression.

**Fig. 4.**
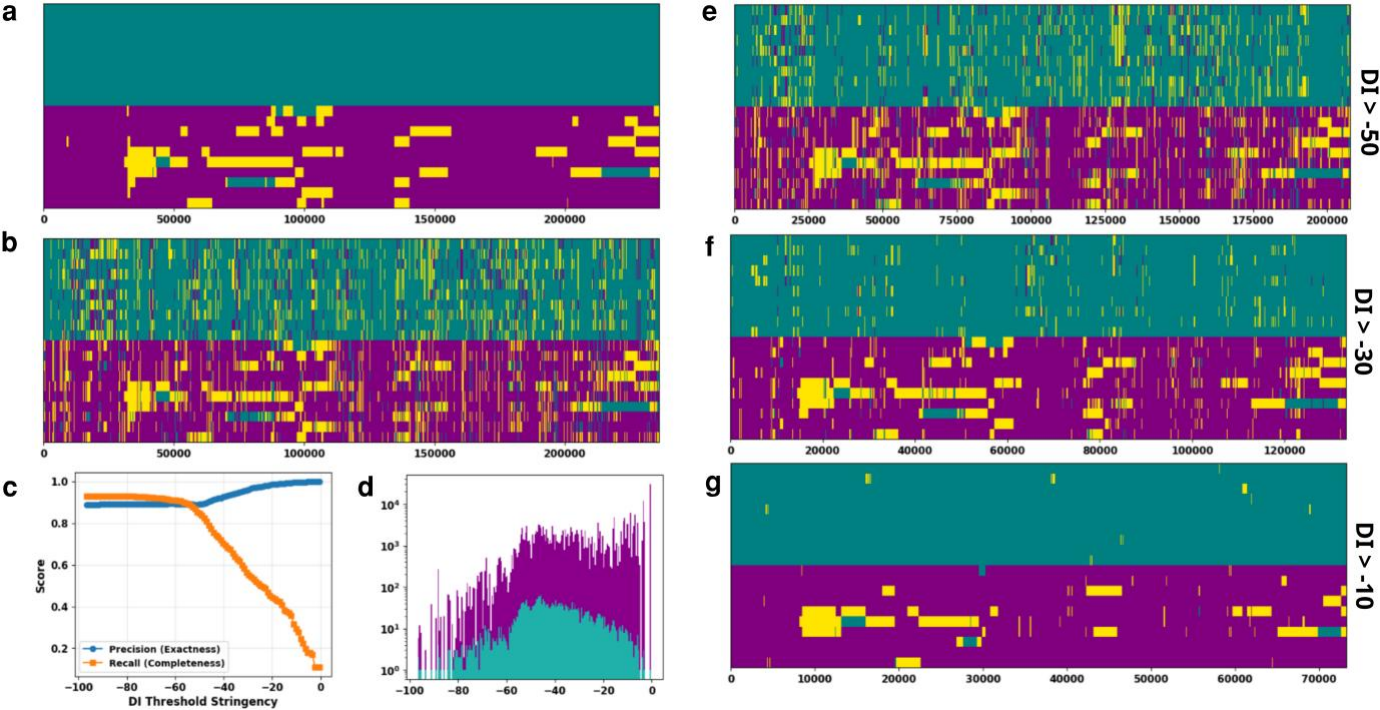
The power to identify introgression tracts. (a) The true genealogy underlying each variant (which may be discordant with the genotype state at a given site). Teal (top) is the donor population and magenta (bottom) is the recipient population forward in time. (b) The polarized simulated variants. (c) Precision and recall as a function of the DI threshold. (d) The corresponding distribution of DI values. Teal shows unique DI scores; magenta includes their multiplicity. (e–g) The markers remaining after applying a minimum DI threshold of −50, −30, and −10, respectively. Here, $T_{\mathrm{split}}=4N_{e}$ generations with $N_{e}=1,000$ in the each the donor, recipient, and ancestor populations.

We test the recall and precision of the *diem* algorithm as a function of the DI threshold in Fig. 4 (see Methods). Note that the recall does not reach 1.0 even when no threshold is applied (the data in Fig. 4b). This is because some variants in the data that correspond to ILS or local variation are discordant with the underlying genealogy at a site (Fig. 4a). By applying increasingly stringent thresholds (Fig. 4e–g), we filter out more of the noise, in effect *cleaning* the data to better distinguish contiguous introgression tracts. However, when the threshold is too high, true introgression tracts may be filtered from the data. As expected, the power to correctly polarize the data and correctly identify introgressed tracts is much higher with high divergence $T_{\mathrm{split}}=8N_{e}$ generations (Supplementary Fig. 2), while low divergence $T_{\mathrm{split}}=2N_{e}$ (Supplementary Fig. 3) makes it more difficult to distinguish introgression from ILS.

## Discussion

Above, we have illustrated the core function of diempy, i.e. genome polarization across a genomic barrier. Population genomic data are informative both about long term barriers and recent admixture. diempy provides both an entry point for exploring such data and additional tools for population genomic analysis. While we have briefly outline some of these, we refer the users to the online tutorial for an in-depth tour.

Most analyses of population genomic variation are necessarily explorative. Even when we wish to test strong prior hypotheses, understanding genome scale variation involves both discovery and the testing of hypotheses. We envisage diempy analysis as an iterative process of fast polarization and browsable visualization, allowing the user to focus analysis clearly on their barriers of interest. We hope that the modular design of diempy will facilitate further development, both for the polarization step and postpolarization analyses. First, the polarization procedure can be generalized to simultaneously identify multiple barriers to gene flow in the samples, reducing the potential bias introduced by exploratory and iterative analyses. While it might be thought that focusing on two bi-allelic genotype states limits the number of estimated barriers to one, it is the conformation space of genotypic states across individuals that determines how many barriers can be distinguished. Second, it is straightforward to change the underlying smoothing kernel or implement alternative approaches to smoothing such as an HMM. Thirdly, an obvious downstream analysis postpolarization is the inference of geographic or genomic clines. Finally, there is a clear theoretical connection between genome polarization and the Ancestral Recombination Graph (ARG) (Hudson 1983), which represents the full genealogical history of a sample.

Each unique variant configuration in the data corresponds to a mutation that occurred on a particular branch in a local tree of the ARG, bi-partitioning the sample. While integrating ARG inference is beyond the scope of this software implementation, exploring how DI values map onto ARG topologies presents a highly promising avenue for future research. We expect variants with the highest DI values—those that most cleanly differentiate groups across a barrier—to map to deep, long internal branches that split the major ancestral clades. Conversely, variants with low DI values likely map to shorter, more terminal branches, or to discordant local topologies resulting from incomplete lineage sorting. Moreover, for any admixture history that involves two panmictic populations, the expected frequency of possible variant configurations is fully described by the joint site frequency spectrum (jSFS) (or the length and span of its corresponding branch types Ralph et al. 2020). Thus, configurations that map to the same entry in the jSFS must have the same (expected) DI. Comparison of the observed DI distribution to expectations under two population histories allows departures from that scenario to be quantified. The practical consequence of developing an ARG-based polarization method would be substantial: instead of polarizing individual variants, researchers could polarize the inferred branches and haplotypes themselves. This would provide a mechanistic framework to definitively distinguish whether a variant with a moderate DI value represents a recent introgression event (a recent branch crossing the barrier) or simply retained ancestral variation (a deep, shared ancestral node). Establishing this quantitative mapping for known admixture histories and developing ARG reconstruction algorithms from *diem*-polarized data remain exciting avenues for the field.

## Supplementary Material

jkag140_Supplementary_Data

## Data Availability

The source code for diempy is available on GitHub at https://github.com/Studenecivb/diemPy and is available for installation via PyPi at https://pypi.org/project/diempy/. The main documentation is provided as a comprehensive tutorial available at https://github.com/DerekSetter/tutorial-DiemPy/ with extensive API documentation (https://diempy.readthedocs.io/). Supplemental material available at G3 online.
