# Supplementary material for: diempy: fast and reference-free genome polarization and chromosome painting
Source: G3 (Bethesda). 2026 Jun 1;16(8):jkag140. doi: 10.1093/g3journal/jkag140 (PMC13439930; doi:10.1093/g3journal/jkag140)
Supplement: Supplementary file 1 [file jkag140_supplementary_data.pdf]

diempy: fast and reference-free genome polarisation and chromosome painting

Supporting Information

Derek Setter[1], Konrad Lohse[1,¶], and Stuart J.E. Baird[*,2,¶]

[1]Institute of Ecology and Evolution, University of Edinburgh, Edinburgh, EH9 3DW, UK
[2]Institute of Vertebrate Biology, Czech Academy of Sciences, Brno, 603 65, Czechia

∗ Corresponding author: Stuart J.E. Baird, Email: stuartjebaird@gmail.com
¶ These authors contributed equally to this work

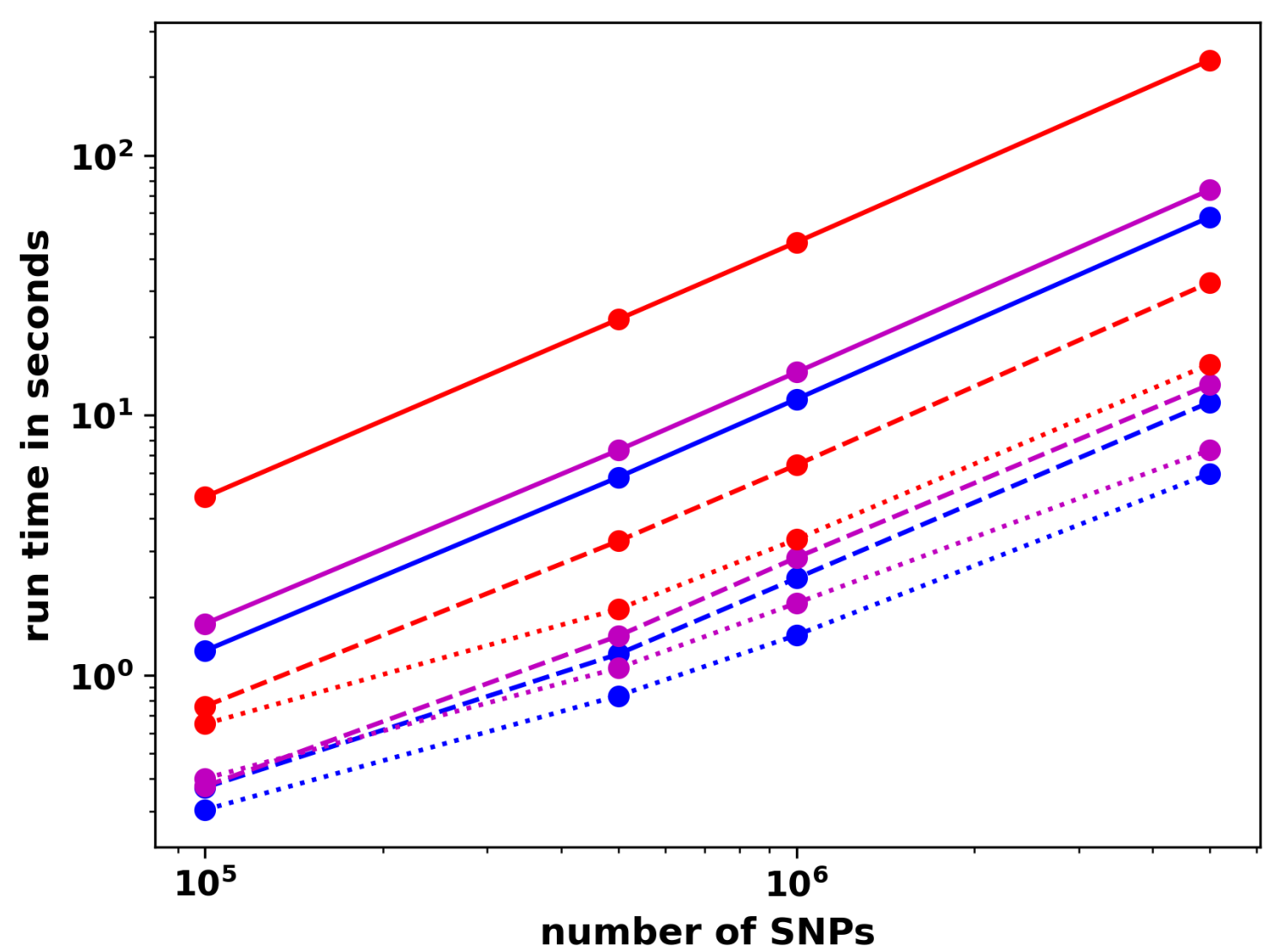

Figure S1: Run time for one iteration of the *diem* EM algorithm in seconds as a function of the number of variants for a sample of 10 individuals (blue), 100 individuals (magenta) and 1000 individuals (red). Solid lines represent sequential processing on a single core. The run time with parallel processing is shown for 5 and 20 cores (dashed and dotted lines respectively).

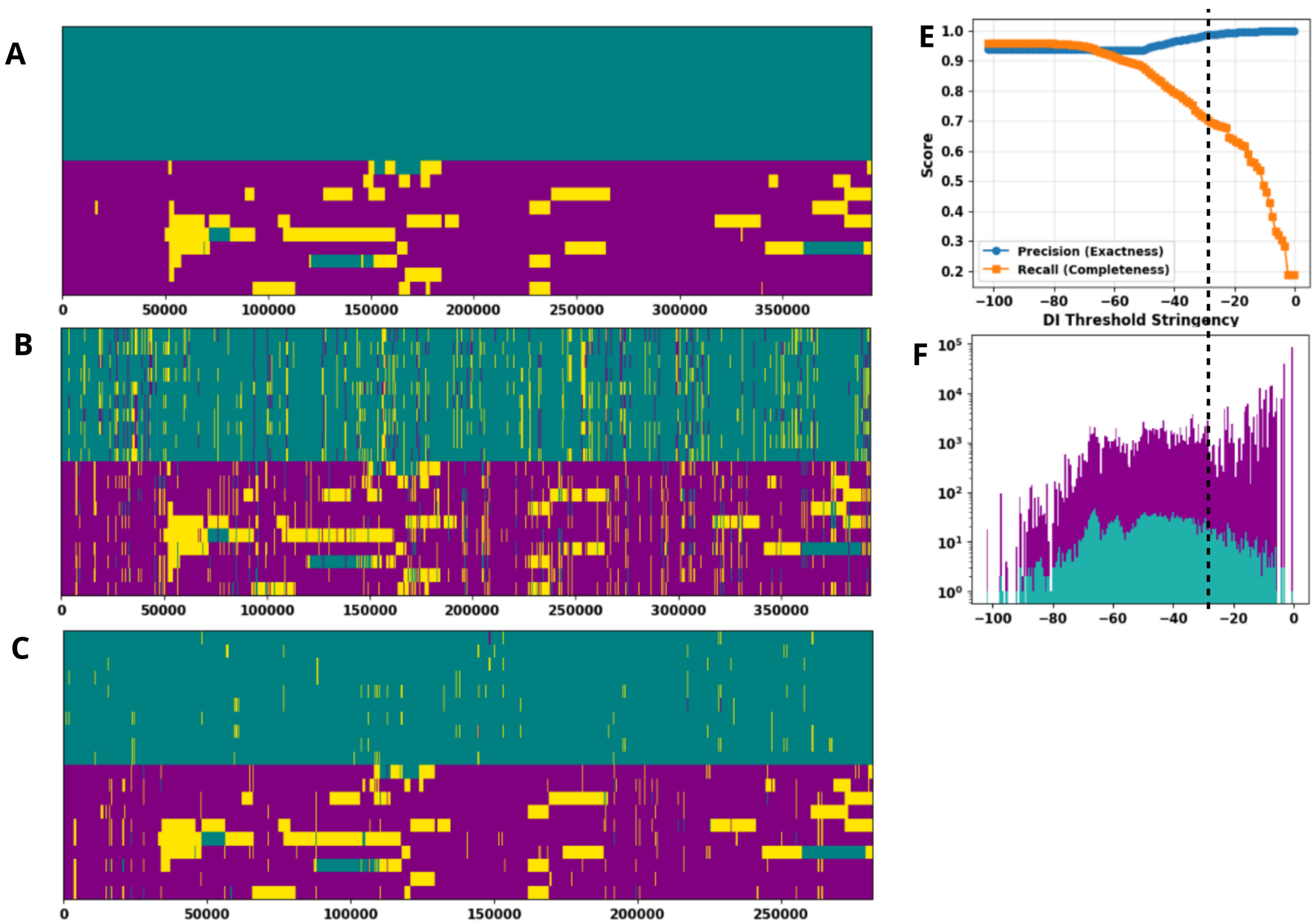

Figure S2: Power to detect introgression with high divergence. The history of migration is identical to Fig. 4 of the main text. Here, the split time $T_{split} = 8N_e$ and there are a greater number of polymorphisms in the dataset. Panel A) the true genealogy underlying each variant. B) The polarized data. C) The data retained after applying a DI threshold of -30. E) The precision and recall as a function of DI threshold. F) The underlying DI distribution.

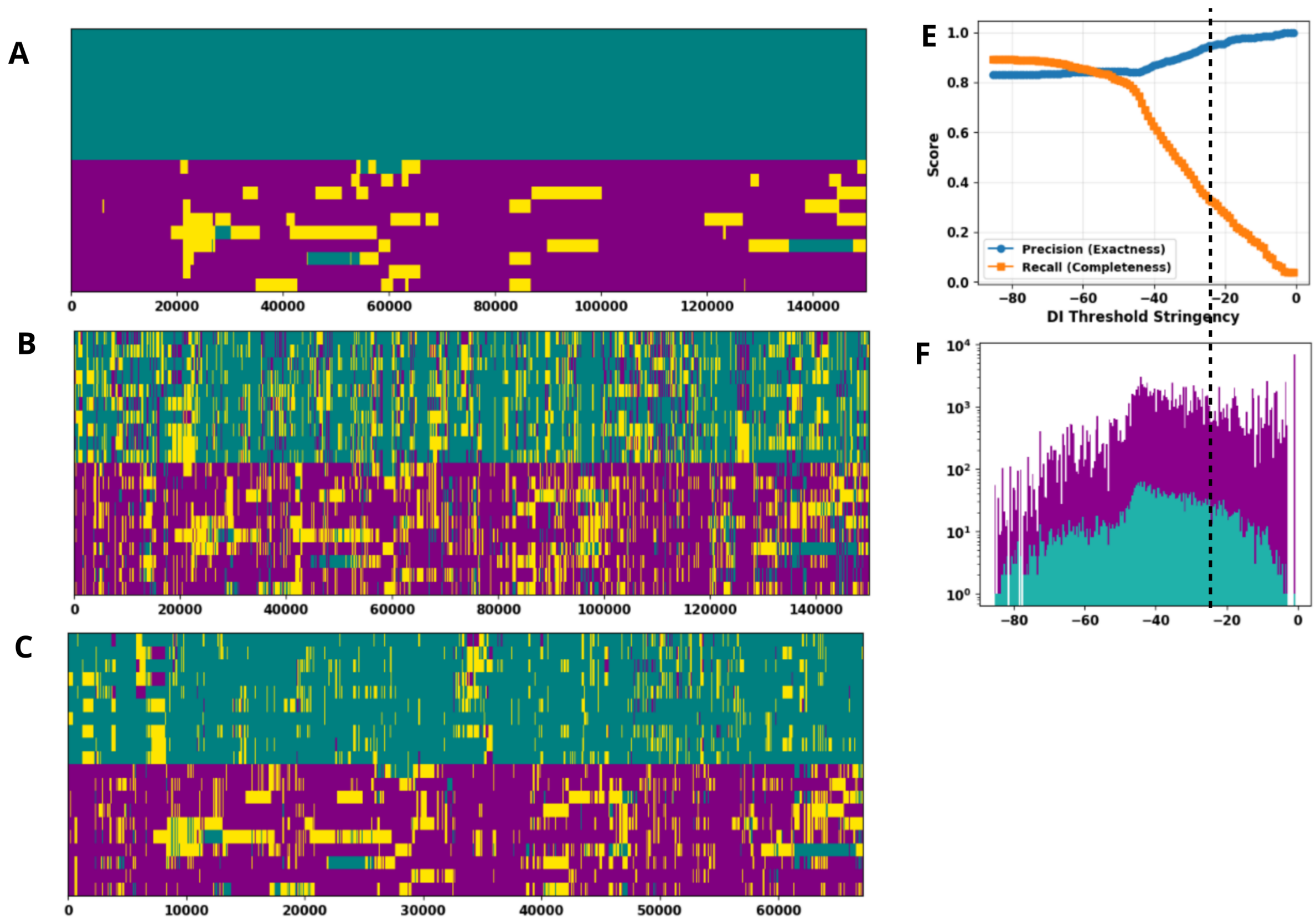

Figure S3: Power to detect introgression with low divergence. The history of migration is identical to Fig. 4 of the main text and Fig. S2 but here the split time $T_{split} = 2N_e$. Panel A) the true genealogy underlying each variant. B) The polarized data. C) The data retained after applying a DI threshold of -30. E) The precision and recall as a function of DI threshold. F) The underlying DI distribution.
